# The circadian clock rephases during lateral root organ initiation in *Arabidopsis thaliana*

**DOI:** 10.1038/ncomms8641

**Published:** 2015-07-06

**Authors:** Ute Voß, Michael H. Wilson, Kim Kenobi, Peter D. Gould, Fiona C. Robertson, Wendy A. Peer, Mikaël Lucas, Kamal Swarup, Ilda Casimiro, Tara J. Holman, Darren M. Wells, Benjamin Péret, Tatsuaki Goh, Hidehiro Fukaki, T. Charlie Hodgman, Laurent Laplaze, Karen J. Halliday, Karin Ljung, Angus S. Murphy, Anthony J. Hall, Alex A. R. Webb, Malcolm J. Bennett

**Affiliations:** 1 Centre for Plant Integrative Biology, School of Biosciences, University of Nottingham, Sutton Bonington Campus, Sutton Bonington, Nottingham LE12 5RD, UK; 2 Department of Comparative and Functional Genomics, Institute of Integrative Biology, University of Liverpool, Liverpool L69 7ZB, UK; 3 Department of Plant Sciences, University of Cambridge, Cambridge CB2 3EA, UK; 4 Department of Environmental Science & Technology, College of Agriculture & Natural Resources, University of Maryland, College Park, Maryland 20742, USA; 5 Institut de Recherche pour le Développement, UMR DIADE, Montpellier 34394, France; 6 Departamento Anatomía, Biología Celular Y Zoología, Facultad de Ciencias, Universidad de Extremadura, Badajoz 06006, Spain; 7 UMR 7265 CEA, CNRS, Université d'Aix-Marseille, Laboratoire de Biologie du Développement des Plantes, Saint-Paul-lez-Durance 13108, France; 8 Department of Biology, Graduate School of Science, Kobe University, Kobe 657-8501, Japan; 9 School of Biological Sciences, University of Edinburgh, Edinburgh EH9 3JH, UK; 10 SynthSys, CH Waddington Building, University of Edinburgh, Edinburgh EH9 3JD, UK; 11 Umea Plant Science Centre, Department of Forest Genetics and Plant Physiology, Swedish University of Agricultural Sciences, Umea SE-901 83, Sweden; 12 Department of Plant Science and Landscape Architecture, University of Maryland, Maryland 20742, USA

## Abstract

The endogenous circadian clock enables organisms to adapt their growth and development to environmental changes. Here we describe how the circadian clock is employed to coordinate responses to the key signal auxin during lateral root (LR) emergence. In the model plant, *Arabidopsis thaliana*, LRs originate from a group of stem cells deep within the root, necessitating that new organs emerge through overlying root tissues. We report that the circadian clock is rephased during LR development. Metabolite and transcript profiling revealed that the circadian clock controls the levels of auxin and auxin-related genes including the auxin response repressor *IAA14* and auxin oxidase *AtDAO2*. Plants lacking or overexpressing core clock components exhibit LR emergence defects. We conclude that the circadian clock acts to gate auxin signalling during LR development to facilitate organ emergence.

## Abstract (web summary)

In *Arabidopsis*, lateral root emergence requires the penetration of overlying tissues by stem cells deep within the root. Voß *et al*. reveal that changes in auxin signalling required for this process are mediated by local rephasing of the circadian clock.

## Graphical abstract

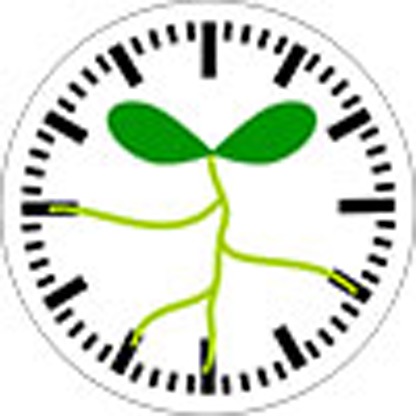

The circadian clock plays a key role in controlling plant growth and development. Genetic studies in the model plant *Arabidopsis thaliana* have revealed that the circadian clock is composed of interacting repressing loops. The core loop consists of the morning loop genes, *LATE ELONGATED HYPOCOTYL* (*LHY*) and *CIRCADIAN CLOCK-ASSOCIATED 1* (*CCA1*), which repress the evening loop gene *TIMING OF CAB EXPRESSION 1* (*TOC1*), which in turn represses both morning and evening loop genes^1^,^2^,^3^,^4^,^5^,^6^ (reviewed in ref. 3).

In plants, the circadian clock is traditionally considered to be cell autonomous but there is emerging evidence of synchronization between the oscillators in individual cells^7^. It has been shown that the plant clock is organ-specific but not organ-autonomous^8^, however, the mechanisms by which circadian rhythms become established or how the circadian clock synchronizes between cells currently represent important areas of research.

Lateral roots (LR) provide an ideal system to study how adjacent populations of plant cells synchronize their growth and development (reviewed in ref. 9). In *Arabidopsis*, LRs are derived from pairs of xylem pole pericycle founder cells located deep within the primary root. These cells undergo several rounds of anticlinal, periclinal and tangential cell divisions to form a new LR primordium^10^. Auxin is a major regulator in this process^9^.To emerge into the soil, a LR primordium first has to break through overlying endodermal, cortical and epidermal cells. This is achieved by employing the hormonal signal auxin to reprogramme overlying cells to undergo cell separation and facilitate the emergence of the new root organ^11^,^12^,^13^.

In this current study, we generated a transcriptomic time course data set of LR development and found that the circadian clock is rephased during LR initiation and that its regulation of auxin-related components acts to control the rate of LR emergence. Disrupting clock function or oscillatory expression of downstream auxin-related targets impairs LR emergence.

## Results

### All core circadian clock genes oscillate in LR primordia

**Figure 1:**
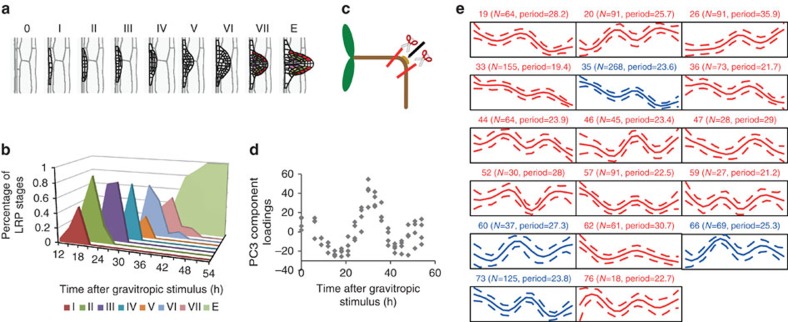
Generation of a lateral root transcriptomic data set. (**a**) Overview of lateral root primordium stages, as described in ref. 10. Image adapted from ref. 14. (**b**) Gravitropic stimulation was used to induce the synchronized initiation of lateral root primordia at the site of root bending in a population of 3-day-old seedlings. Lateral root primordium stages (from I to VIII according to previous descriptions from ref. 10) were determined every 3 h from 6 to 54 h post gravity induction and are represented here as a percentage of the total number of observed lateral root primordia at each time point^14^. (**c**) Root bends were microdissected for each of those 18 time points and used for RNA extraction and subsequent microarray analysis (*N*=450 per time point, 4 independent biological replicates). All seedlings were grown in constant light conditions, without previous entrainment. Image is adapted from ref. 14. (**d**) Principal component analysis of the lateral root data set revealed an oscillating component in the third principal component (PC3). (**e**) When differentially expressed genes were clustered, 17 clusters showed an oscillatory pattern. The estimate of the period length (h) and the number of genes in each cluster (*N*=*x*) is indicated above each cluster. Clusters with blue lines comprise core circadian clock genes. Expression intensities are on a log2 scale. The mean of the clusters is given by the solid line and the dotted lines show the mean±2s.d.

**Figure 2:**
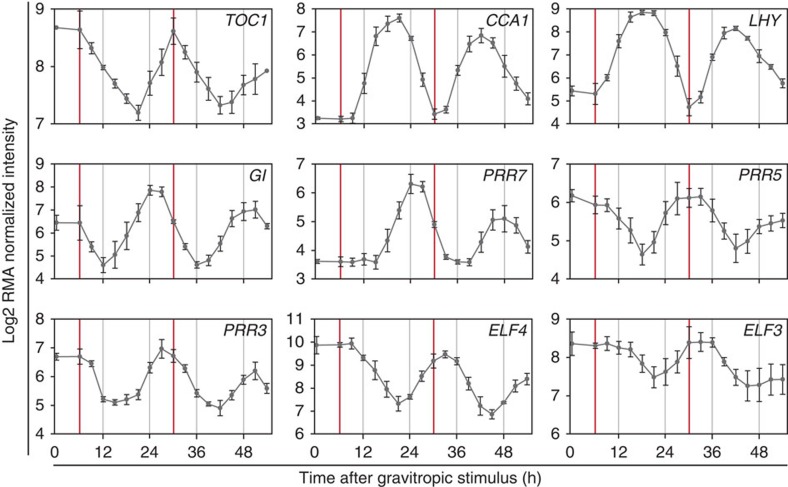
Circadian oscillations in the lateral root time course. RNA expression patterns of the circadian clock genes *CCA1*, *LHY*, *GI*, *TOC1*, *PRR7*, *PRR5*, *PRR3*, *ELF4* and *ELF3*, all of which oscillate in the lateral root data set. Expression intensities from the lateral root microarray data are on a log2 scale. Red vertical bars indicate peak times of *TOC1* expression. *N*=4. All seedlings were grown in constant light conditions, without previous entrainment. The mean of the gene expression is given by the solid line and the error bars show the mean±2s.e.

To identify novel genes and mechanisms regulating LR development, we initially generated a transcriptomic time course data set covering every stage of LR development. We previously showed that, following a 90° gravitropic stimulus, LRs develop in a highly synchronized manner at the outer edge of a bending root (Fig. 1a,b)^14^. In *Col-0* roots, under our growth conditions, stage I LR primordia are detected at 15 h post gravity induction (pgi) and LRs emerged 42 h pgi (Fig. 1a,b). We took advantage of this regular LR primordium development by microdissecting root bends every 3 h from 6 to 54 h pgi (Fig. 1b,c). For this, *Col-0* seedlings were grown without previous entrainment in constant light conditions for 3 days, before the gravity stimulus was applied. From four independent biological replicates of each time point, Affymetrix transcript abundance data were generated, encompassing every stage from pre-initiation to post-emergence (Fig. 1b; microarray data are publicly available at ArrayExpress (www.ebi.ac.uk/arrayexpress/) under the accession number E-MTAB-2565; all data shown in this manuscript are averages from those four replicates). Initial quality control testing confirmed the ability of the data set to identify LR development regulatory genes. We examined the transcript abundance of several known key LR regulators: *AUXIN RESPONSE FACTOR* (*ARF*) *19*, *LATERAL ORGAN BOUNDARIES DOMAIN* (*LBD*) *16*, *18* and *29*, *SHORT HYPOCOTYL 2* (*SHY2*), *GATA TRANSCRIPTION FACTOR 23* (*GATA23*), *CYCLIN B1;1* (*CYCB1;1*) and *LIKE AUX1 3* (*LAX3*) (Supplementary Fig. 1), which were all significantly upregulated at previously published time points^13^,^15^,^16^,^17^,^18^,^19^. In total, >8,000 genes were differentially expressed across all time points and principal component (PC) analysis revealed an oscillatory pattern in the third PC with a period of ∼24 h (PC3; Fig. 1d)^20^. Clustering of transcript abundance profiles generated a set of 77 clusters, revealing a broad range of gene mRNA patterns during LR development (Supplementary Fig. 2 and Supplementary Data 1). Consistent with PC3, 17 clusters exhibited an oscillatory pattern with a period ranging from 19.4 to 35.9 h (Fig. 1d,e; Supplementary Fig. 2), and four oscillating clusters comprised well-characterized core circadian clock genes (blue in Fig. 1e and Supplementary Fig. 2; cluster 35 contains *ELF3*, cluster 60 *GI* and *PRR7*, cluster 66 *CCA1* and *LHY*, cluster 73 *TOC1*, *PRR5*, *PRR3* and *ELF4*; the individual transcript abundance patterns of these nine core circadian clock genes are shown in Fig. 2).

To validate that the LR transcriptome displays circadian oscillations under free-running conditions, we analysed the data set using the JTK_CYCLE algorithm^21^. Rhythmic components were identified in the mRNA patterns of 1,575 genes, including all the core circadian clock genes, with an average period length of 25.3 h (Supplementary Data 2). As such a large proportion of genes were oscillating close to a 24-h period in the data set, we investigated the behaviour of the set of nine core circadian clock component genes in more detail (Fig. 2). All nine genes clearly showed oscillatory transcript abundance, and the period of rhythms in *LHY* and *CCA1* mRNAs in the LR is similar to the period of their expression measured in whole roots^8^ (Supplementary Data 2). Morning loop genes including *CCA1* and *LHY* were oscillating in anti-phase to the evening loop gene *TOC1* (indicated by vertical red lines in Fig. 2), indicating that we are observing a fully operating circadian clock in LR development.

It has been previously described that only the day loop is fully operating in *Arabidopsis* roots, synchronized by photosynthetic signals derived from the shoot circadian oscillator^8^. The most likely difference with our observations is due to different growth conditions. In the cited work, plants were grown hydroponically in light-tight boxes. Therefore, under constant light conditions, the leaves were in constant light but the roots were in constant darkness. In our experiments, both roots and leaves are exposed to constant light. Another difference is that whole roots were analysed^8^, whereas here we follow the development of LR primordia. When entrained in light–dark cycles the day-loop reporter *TOC1:LUC* oscillates in primary and lateral roots while in constant dark conditions waves of circadian oscillations travel from the root tip upwards^22^. This principle is very similar to that of a non-circadian oscillation of auxin responsive gene expression, such as *ARF7*, along the longitudinal axis of the root that determines the priming of LR primordia sites^23^. In contrast, here we investigate the establishment of fully operating circadian rhythms, including all evening loop components, and coordination of circadian signals as new LR primordia develop in growing roots.

### The circadian clock rephases during LR development

**Figure 3:**
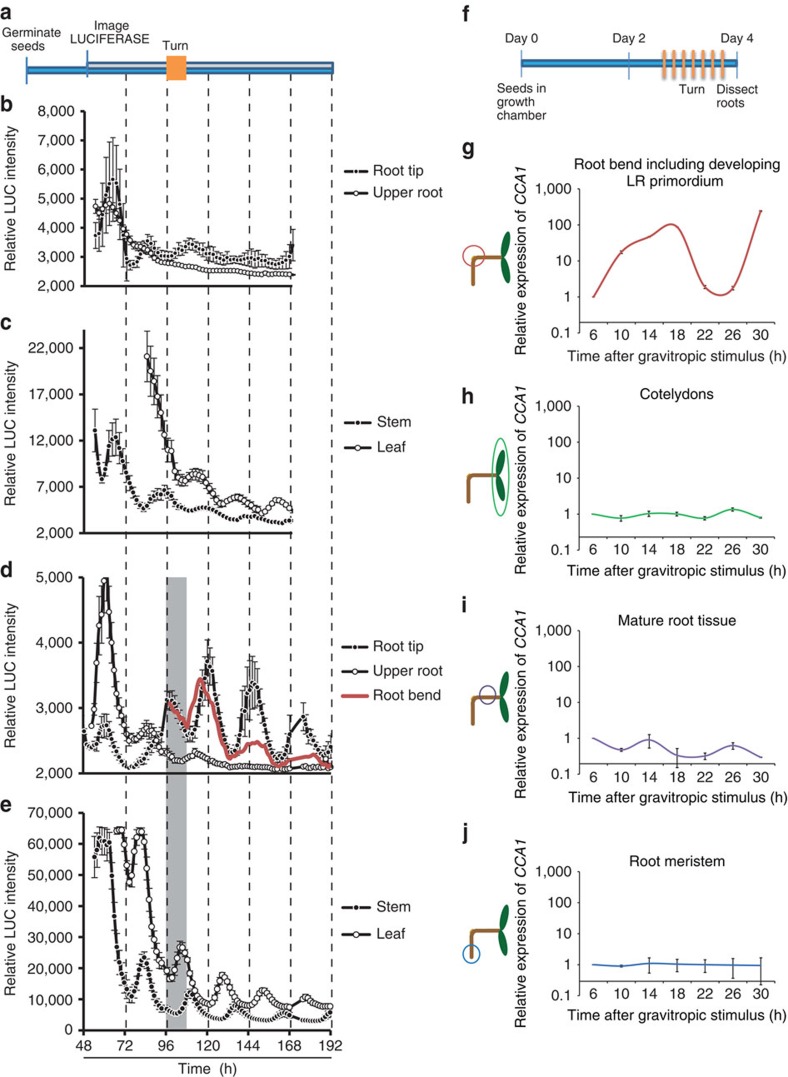
Gravitropic stimulation regulates the circadian clock in the root bend. (**a**) Luminescence levels of *TOC1:LUC* plants were continuously monitored. *TOC1:LUC* seedlings were grown vertically and imaged after the seeds were placed on plates. (**b**,**c**) Luminescence of control seedlings without gravitropic stimulus was monitored in: root tip and upper root (**b**), stem and leaf (**c**). (**d**,**e**) In contrast to **b**,**c** after 4 days a 90° gravitropic stimulus, as indicated by the grey section, was applied for 12 h. After that period, the plate was rotated back to original start position. (**b**–**e**) Error bars indicate s.e.; *N*=2. (**f**) From seedlings that have been exposed to a gravitropic stimulus at different ages, various tissues (root bend (**g**), cotyledons (**h**), upper root (**i**) and root meristem (**j**); indicated by circles) were dissected at the same time/age of the seedling (shown in **f**). (**g**–**j**) RNA was prepared and RT–qPCR was used to measure the levels of *CCA1* mRNA. Only the root bends show oscillation of *CCA1* expression. Error bars indicate s.d.; *N*=4.

The observed circadian oscillations in the LR data set can be explained by two different hypotheses: (H1) circadian oscillations in the root bend are initiated at germination^24^ or, (H2) the root gravitropic stimulus or LR primordium initiation rephases the circadian clock in the root bends, possibly independent of other tissues. To distinguish between these hypotheses, we applied a gravitropic stimulus to a *TOC1:LUC* reporter line, and monitored luminescence from germination onwards in continuous light conditions (Fig. 3a–e). When no gravitropic stimulus was applied, root tip and stem initially oscillated in phase, but oscillations dampened after 2 days in all parts of the root (Fig. 3b), while oscillations still persist in leaf and stem tissue (Fig. 3c). After gravity stimulation, robust oscillations became visible in the root bend and root tip tissues and persist over the course of the experiment. Regions above the bend still dampened quickly, similar to the control data in absence of a gravitropic stimulus. When comparing the region at which root bending occurs to the robustly rhythmic root tip, it appears that the bending initially triggers an earlier phase (1–3 h) and also a longer periodicity, suggesting that this region at the root bend is behaving differently to other parts of the root tissue and other parts of the plant (Fig. 3d,e). However, 48 h after gravity stimulation the phases of the root bend and root tip synchronize. This finding can be explained by recent data showing that the vascular tissues of cotyledons, where the circadian clock is distinct and more robust than in mesophyll tissues, can regulate gene expression as well as physiological responses in the neighbouring mesophyll tissue^7^. In the case of a root bend, 48 h after gravity stimulation the new LR has emerged (Fig. 1b) and its vascular tissues have differentiated. We hypothesize that this maturation of the LR vascular tissues could explain the rephasing of the LR clock to the primary root clock. Irrespective, these data indicate that the LR circadian clock initially oscillates in a phase independent of the circadian clock of other root tissues.

To validate that the LR circadian clock does indeed oscillate in a phase independent of the circadian clock in other tissues, we conducted another experiment. Again, all seedlings were grown in continuous light conditions, without previous entrainment, consistent with the growth conditions in the previous experiments. To prove that both the morning and evening loops of the circadian clock rephase during LR development, we measured expression levels of the morning loop gene *CCA1* in this experiment. In contrast to the microarray experiment (Fig. 1), all seedlings were dissected at the same age (same duration in the growth chamber), but gravity-stimulated at different ages post germination (Fig. 3f). From these plants, cotyledons, root bends, meristems and root sections above the bending region were dissected and *CCA1* mRNA levels were measured by quantitative reverse transcription PCR (qRT–PCR; Fig. 3g–j). If the gravistimulus did not change the circadian clock phase in the investigated tissue, one would expect a flat expression curve, as all samples would be harvested in the same phase of oscillation. However, if the gravistimulus leads to rephasing of the oscillator, one would observe *CCA1* oscillations when plotting the data against ‘time after gravitropic stimulus’. Our results reveal that *CCA1* transcript abundance starts to oscillate in the root bend itself (Fig. 3g), while in the other sampled tissues (Fig. 3h–j) the mRNA profile of *CCA1* was flat, confirming that the circadian clock rephases at the root bend after the gravitropic stimulus, independent of other plant tissues.

### *TOC1* is auxin-inducible during LR initiation

**Figure 4:**
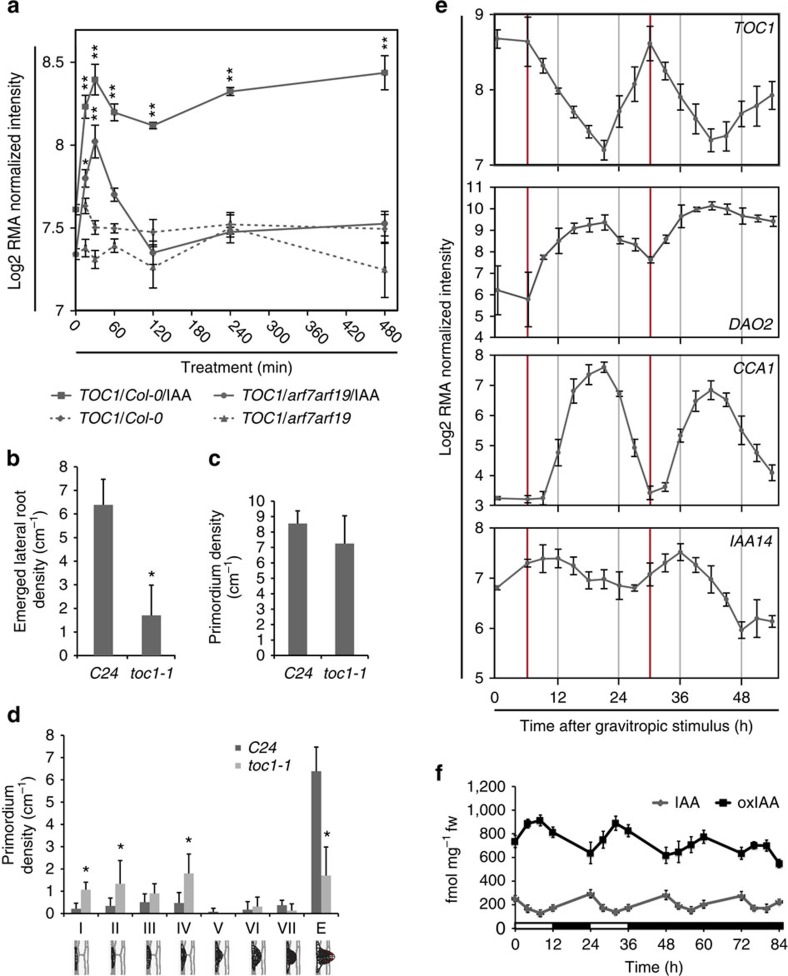
Lateral root phenotypes of circadian clock mutants. (**a**) *TOC1* gene expression in the basal meristem is induced in response to auxin treatment of whole seedlings in *Col-0*, as well as *arf7arf19* mutant plants. Expression intensities are on a log2 scale and are based on transcriptomics data with an ATH1 chip (Affymetrix), three independent biological replicates each. Error bars indicate s.d.; asterisks indicate a significant difference (*P*<0.05, Student’s *t*-test) from wildtype. (**b**–**d**) All seedlings were grown in constant light conditions, without previous entrainment. *N*=20 for all experiments. Error bars indicate s.d.; asterisks indicate a significant difference from wildtype. (**b**) Emerged lateral root density of 10-day-old *C24* and *toc1-1* seedlings in cm^−1^ revealed a strong phenotype for *toc1-1*. (**c**,**d**) Ten-day-old seedlings were fixed and the primordium density of different developmental stages (stage I to emerged (E)) of wild-type (*C24)* and *toc1-1* mutant seedlings in cm^−1^ was determined. (**e**) Comparison of oscillating gene expression patterns from the lateral root transcriptomic time course of the circadian clock genes *CCA1* and *TOC1* and auxin signalling genes *IAA14* and *AtDAO2*, demonstrate gating of auxin signalling by the circadian clock during lateral root primordium development. Expression intensities are on a log2 scale. Red vertical bars indicate peak times of *TOC1* expression. All seedlings were grown in constant light conditions, without previous entrainment. The mean of the gene expression is given by the solid line and the error bars show the mean±2 s.e. *N*=4. (**f**) IAA and oxIAA accumulations exhibit circadian rhythms in roots under free-running conditions. Seedlings were germinated and entrained under 12 h light/dark cycle before transfer to constant darkness. Tissue was harvested starting at subjective dawn for 5-day seedlings. The experiment was repeated three times. White bars, light period; dark bars, dark period. Error bars indicate s.d.; *N*=3.

At the start of the LR time course, transcripts of the morning genes *CCA1*, *LHY* and *PRR7* are low, whereas mRNAs of afternoon and evening expressed genes *PRR5*, *PRR3*, *GI*, *TOC1*, *ELF3* and *ELF4* are high (Fig. 2). The first significant changes in circadian clock transcript abundance are a rapid decline of *TOC1* and *GI* versus a small increase in *LHY* mRNA abundance from 9 h pgi (Fig. 2). All other circadian clock genes start to oscillate later (Fig. 2), suggesting that *TOC1*, *GI* and/or *LHY* genes regulate rephasing of circadian clock oscillations in LRs. This pattern of transcript abundance is similar in dry seeds before imbibition^24^, which is believed to synchronize and set the phase of the clock in *Arabidopsis*^25^. Since *TOC1*, *GI* and *LHY* are candidates for regulating circadian clock rephasing, and given that the plant hormone auxin triggers LR initiation, we investigated whether their expression is regulated by this hormone. Seedling roots were first treated with auxin (indole-3-acetic acid (IAA)) and then apical meristem and elongation zones (the latter containing the basal meristem from which new LR primordia originate) were microdissected, then the RNA isolated and profiled (as described for root bends). In the transcriptomic data generated using wild-type (WT) (*Col-0*) root material, *TOC1* transcript abundance was rapidly induced nearly twofold 15 min after auxin treatment in the basal meristem where LRs are primed (Fig. 4a), while in the root meristem the induction is nearly absent (Supplementary Fig. 3a). To validate this observation, we transcript profiled equivalent auxin-treated root zones in the *arf7arf19* mutant (Fig. 4a and Supplementary Fig. 3a). ARF7 and ARF19 are two (of a total of five) auxin response (transcription activating) factors in *Arabidopsis* that are required for LR initiation since *arf7arf19* mutants do not form LRs^12^,^15^,^16^. As the auxin response pathway and LR development are disrupted in *arf7arf19*, we employed this double mutant background as a control for the auxin transcriptomic experiment. We observed that while ARF7 and ARF19 are not required for the initial rapid auxin-dependent upregulation of *TOC1*, they were essential for the maintenance of high *TOC1* transcript levels (Fig. 4a). In contrast to *TOC1* and *LHY*, root transcript abundance was not significantly changed by auxin (Supplementary Fig. 3d,e). It has been demonstrated that high auxin concentrations can affect, but do not rephase the circadian clock^26^ and that pulses of *CCA1 or LHY* expression can shift the phase of circadian rhythms, but pulses of *TOC1* expression do not^27^. In addition, *TOC1* expression decreased after IAA treatment^26^. However, these observations were made in whole seedlings. As other major differences have been described for root and shoot circadian clock regulation^8^, we cannot rule out that this mechanism might be different in roots or LR primordia in particular. The observed auxin induction of *TOC1* gene expression cannot be seen in other auxin and LR time course data sets^28^,^29^,^30^,^31^. However, the time points sampled do not have the resolution to pick up the rapid induction of *TOC1* (within 15 min). In addition, whole seedlings or whole roots were sampled for those data sets, most likely resulting in a dilution of *TOC1* induction, which is specific to the root basal meristem. Based on this data, we suggest that auxin controls the induction and maintenance of *TOC1* expression, representing a novel role for auxin in LR circadian clock regulation.

### The circadian clock is required for LR development

To determine whether a fully operating circadian clock is required for normal LR development, we compared LR densities in single mutants and overexpressing lines of the three core circadian clock genes *CCA1* (*cca1-1, CCA1-OX*), *LHY* (*lhy-21, LHY-OX*) and *TOC1* (*toc1-1, TOC1-OX*) (Fig. 4b, Supplementary Figs 4a,c and 5b–e)^32^,^33^,^34^. For phenotyping, all plants were grown in continuous light conditions, without previous entrainment, consistent with growth conditions from the previous experiments. The strongest reduction in emerged LR and LR primordium density was found in *toc1-1*, *CCA1-OX* and *LHY-OX* lines (between 30 and 60%), whereas other lines displayed weaker LR developmental phenotypes (Fig. 4b, Supplementary Figs 4b and 5b). While the absence of LR phenotypes in *TOC1-OX* and *lhy-21* mutants seems puzzling on the first glance, a similar variety of phenotypic occurrence has been described for various other circadian clock-associated phenotypes. For example, *CCA1-OX*, *LHY-OX*, *elf3*, *prr7* and *prr9* exhibit long hypocotyl phenotypes, while the *toc1-1* mutant does not^3^,^35^. Similar phenotypic discrepancies among circadian clock mutants have been shown in the control of flowering timing^3^. In addition, we attribute the phenotypic differences between these lines to *toc1-1* being a semi-dominant missense allele (in its DNA-binding domain)^36^ that disrupts the mutant *toc1-1* protein binding to promoters such as *CCA1* to repress their expression^4^, while CCA1 and LHY overexpression has been reported to be able to reset the phase of the circadian clock^27^. To determine whether this phenotype arose as a result of a defect in LR initiation or development, we profiled the stages of LR primordia in 10-day-old seedlings in *toc1-1*, *LHY-OX* and *CCA1-OX* and compared them to their respective WTs. The proportion of *toc1-1* LR primordia between stages I and IV was increased threefold compared with the WT control (*C24*, Fig. 4d), although the overall primordia density, including emerged LRs, was similar (Fig. 4c). *LHY-OX* lines also displayed a threefold increase in stage IV primordia (Supplementary Fig. 5c), but the overall primordia density was still lower than WT (Supplementary Fig. 5b). *CCA1-OX* lines did not display a strong LR primordium phenotype. These data suggest that disrupting the circadian clock leads to strong defects in LR development or emergence, but not initiation (Fig. 4b–d, Supplementary Figs 4 and 5). The transition from stage II to V is an important phase in LR development, since the Casparian strip, a tough lignified barrier that effectively ropes together endodermal cells overlying new LRs, needs to be broken to allow the new root organ to emerge^37^. Auxin is a key signal promoting LR emergence^11^,^38^,^39^. This hormone is transported from newly initiated LR primordia towards cells in overlying tissues, where it triggers the expression of genes including its own influx carrier, *LAX3*, and the cell-wall remodelling enzyme polygalacturonase^11^,^14^,^40^.

### The LR circadian clock gates auxin levels and response

Given the LR development or emergence defects exhibited by mutant and overexpresser lines of core clock components, we examined whether the circadian clock may gate auxin signalling during LR development. We observed that 49 auxin-related genes display circadian oscillations during LR development under our experimental conditions^21^ (see Supplementary Data 3 for oscillating auxin-related genes). Among these auxin-related genes is *IAA14* (Fig. 4e), which is a key regulator of LR initiation^41^ and emergence^9^. Significantly, expressing *IAA14* under the regulation of the *35S* promoter blocks LR emergence^42^. Hence, replacing the native *IAA14* gene’s oscillatory pattern (Fig. 4e) with a constitutive mode of expression in *35S::IAA14* transgenic seedlings disrupts LR emergence^42^. IAA14 functions as a transcriptional repressor that interacts directly with ARF7 and ARF19 proteins. Hence, regulating *IAA14* expression in a circadian manner (Fig. 4e) would serve to gate the activity of the IAA14/ARF7/ARF19 auxin response module and their downstream genes that include direct targets *LBD16* and *LBD29* (refs 43, 44). Consistent with this model, LBD29, and to a lesser extent LBD16, are among the auxin-related genes whose mRNAs display circadian oscillations during LR development in our transcriptomic data set (Supplementary Fig. 1 and Supplementary Data 3).

*AtDAO2* (*AT1G14120*) represents another strongly circadian oscillating auxin-related mRNA (Fig. 4e, Supplementary Data 3). *AtDAO2* is orthologous to the rice DAO enzyme mediating degradation of the major plant auxin IAA to its biologically inactive form oxIAA^45^. Metabolic profiling of root tissues revealed that oxIAA levels display strong circadian oscillations under free-running conditions (after initial entrainment for 2 days), and which increases during the subjective day coincident with elevated *AtDAO2* transcript abundance (Fig. 4f). In parallel, free IAA levels oscillate in anti-phase with *AtDAO2* and oxIAA (Fig. 4e,f). The circadian patterns of *AtDAO2* and IAA levels (Fig. 4e,f) would lead to the gating of auxin-inducible gene expression such as *IAA14* during root development. Moreover, the turnover of the IAA14 protein is also auxin dependent^46^,^47^ creating further opportunities to gate auxin response activity during LR development. Indeed, slowing the auxin-dependent dynamics of IAA14 degradation was recently demonstrated to cause a quantitative reduction in LR emergence^48^.

*AtDAO2* exhibits a circadian oscillation in transcript abundance similar to *CCA1* but in anti-phase to *TOC1* (Fig. 4e). *AtDAO2* mRNA is initially induced one time step before *CCA1*, suggesting that its circadian expression pattern is not dependent on CCA1. The *AtDAO2* promoter contains nine TOC1 (T1ME) binding motifs and TOC1 has recently been reported to be a transcriptional repressor^4^. In addition, *AtDAO2* induction coincides with *TOC1* transcript abundance dropping and *vice versa*, suggesting that *AtDAO2* is negatively regulated by TOC1 (Fig. 4e). TOC1 functioning to directly repress *AtDAO2* expression could explain why the *toc1-1* mutant exhibits such a severe LR phenotype (Fig. 4b–d, Supplementary Fig. 4) since expression of many other auxin regulated genes (in addition to *AtDOA2*) would be uncoupled from the circadian clock.

## Discussion

We conclude that a fully operating circadian clock is necessary during LR emergence. Endo *et al*.^7^ recently proposed that circadian clocks of different tissues perform distinct functions. Consistent with this, *AtDAO2* and 14 other auxin-related genes (including *LAX3)* oscillating in our LR data set do not exhibit circadian changes in whole seedlings^26^ or cotyledons^7^ (Fig. 4e, Supplementary Fig. 1, Supplementary Data 3). Our results suggest that the circadian clock functions like a developmental ‘metronome’ during LR development, causing the expression of genes such as *AtDOA2* and *IAA14* to gate auxin responses in cells overlying new primordia, and thereby coordinate organ emergence. But why do the circadian clocks of cells in the vicinity of new LR primordia have to be rephased compared with other root tissues? One likely explanation is that cells within and surrounding new LR primordia need to independently regulate their hydraulic properties in a manner distinct to other root tissues to facilitate organ emergence. We recently demonstrated that auxin channelled via the LR primordium into overlying cells functions to repress the expression of genes encoding water channels termed aquaporins, creating complex spatio-temporal changes in hydraulic properties necessary for organ emergence^14^. In contrast, in other root tissues the circadian clock regulates aquaporin expression, resulting in diurnal oscillations in root water uptake^49^,^50^. Rephasing the circadian clock in cells within the new LRP and surrounding tissues would, in effect, transiently hydraulically isolate them from these other diurnal oscillations in root water uptake. Nevertheless, we observed that the circadian phases of the newly emerged lateral root primordium (LRP) and root tip eventually synchronize and hypothesize that the maturation of the vascular tissues in the new root organ could explain this rephasing (akin to the recently proposed role of the vasculature in cotyledons^7^).

## Methods

### Growth conditions and plant material

WT, mutants and reporter lines were grown vertically on sterile 12 × 12 cm square Petri dishes on ½ MS (Murashige and Skoog; pH 5.8) at 23 °C under continuous light (150 μmol m^−2^ s^−1^). For RNA preparation, transcriptomic and phenotypic analysis, LR induction was performed on 3-day-old *Col-0* seedlings grown vertically by rotating the plates by 90°. All mutant lines were obtained from the European *Arabidopsis* Stock Centre (NASC).

### RNA extraction for LR transcriptomic data and qRT-PCR

Total RNA was extracted from roots using Qiagen RNeasy plant mini kit with on-column DNAse treatment following the manufacturer’s recommended protocol (RNAse-free DNAse Set, Qiagen, Crawley, UK). RNA samples were quantified using a Nanodrop ND100 spectrophotometer (Nanodrop, Wilimington, USA).

### Transcriptome analysis of LR development

Four biological replicates from separate pools of seeds (*Col-0*) were stratified for 2 days at 4 °C, before transfer to growth chambers. For every time point, root bends of ∼400 seedlings were microdissected under a binocular microscope and frozen in liquid nitrogen immediately on harvesting as described in ref. 14. In addition to the root bends, a mature root segment located between the bend and the shoot was harvested at 9 h after an inductive gravitropic stimulation to serve as a reference of non-gravitropic-stimulated root tissues devoid of developing LRP (time point 0 in the data set; for more details about timing of sampling see Supplementary Table 1). RNA labelling and hybridization to Affymetrix ATH1 arrays were performed by NASC.

Data were normalized as described in ref. 17. Error bars indicate s.d.; asterisks indicate a significant difference from WT. For timings of plant growth and root sampling see Supplementary Table 1, which applies to all four replicates. Microarray data are available in the ArrayExpress database (www.ebi.ac.uk/arrayexpress) under accession number E-MTAB-2565.

### Transcriptome analysis of WT and *arf7arf19*

Three biological replicates from separate pools of seeds (*Col-0* and *arf7arf19*) were stratified for 2 days at 4 °C, before transferring to growth chambers for 6 days. Sterile 9 × 9 cm square sections of 100 μm nylon mesh (Clarcor) were placed onto the media surface before sowing to facilitate root dissection and harvesting of cut sections. Plates were then transferred to tanks containing 3 l of 0.5 × MS media and allowed to acclimate for 24 h and then to tanks containing 3 l of 0.5 × MS media supplemented with either 30 μl 100% ethanol or 30 μl of 0.1 M IAA in 100% ethanol (1 μM final IAA concentration) for 0, 15, 30, 60, 120, 240 or 480 min.

For each biological replicate, plants were grown and ∼50 roots were dissected into 2 sections; the meristem (from the root tip to the top of the LR cap, ∼350 μm from the tip) and the basal meristem zone (from the top of the LR cap to the first visible root hair bulge, ∼850 μm from the shootward boundary of zone 1). Dissected samples were immediately frozen in liquid nitrogen. RNA was extracted using the Qiagen MicroRNA Kit following the manufacturers recommended protocol (Qiagen) and quantified using a Nanodrop ND100 spectrophotometer. RNA labelling and hybridization to Affymetrix ATH1 arrays were performed by NASC.

Data were normalized as described in ref. 17. Transcriptomics data used in these experiments have been made available through ArrayExpress (www.ebi.ac.uk) with accession number E-MEXP-1354.

### qRT–PCR

Poly(dT) complementary DNA (cDNA) was prepared from 2 μg total RNA using the Transcriptor first strand cDNA synthesis kit (Roche). Quantitative PCR was performed using SYBR Green Sensimix (Quantace) on a Roche LightCycler 480 apparatus. PCR was carried out in 384-well optical reaction plates heated for 1 min to 95 °C, followed by 40 cycles of denaturation for 5 s at 95 °C, annealing for 8 s at 62 °C and extension for 30 s at 72 °C. Target quantifications were performed with the specific primer pairs described in Supplementary Table 2. Expression levels were normalized to *ACTIN*. All qRT–PCR experiments were performed in quadruplicates and the values represent mean±s.e.m.

### LR analysis and microscopy

Total number and stages of LRP were counted using primary roots cleared by immersion in 20%(v/v) methanol/4% (v/v) hydrochloric acid at 57 °C for 20 min, followed by immersion in 7% (w/v) NaOH/60% (v/v) ethanol at room temperature for 15 min. Roots were then rehydrated for 5 min each in 40%, 20% and 10% (v/v) ethanol and mounted in 50% (v/v) glycerol on glass microscope slides and were imaged using a Leica differential interference contrast optics microscope.

### Luciferase imaging

The *TOC1:LUC* reporter line was produced as part of the ROBuST project in a *Col-0* background. Seedlings were grown on vertical ½ MS plates at 23 °C under continuous light. LR induction was performed on 3-day-old *Col-0* seedlings grown vertically by rotating the plate by 90°. About 12 h later, the plate was rotated back to original start position. Luciferase imaging was done as described in ref. 51. Analysis of data was done using Imaris (Bitplane).

### IAA/oxIAA measurements

*A. thaliana* *Col-0* seeds were sown on ¼ MS, 0.5% sucrose pH 5.7 phytagar plates, and stratified at 4 °C for 48 h. The seeds were transferred to a Conviron growth chamber, 120 μE, 12 h day/night. Seedlings were harvested starting at day 5. For free-running circadian experiments, samples were conducted as in the 12 h day/night experiments except that the growth chamber was shifted to 24 h darkness for the period described. Roots were collected, briefly patted dry, weighed in 1.5 ml centrifuge tubes and frozen in liquid nitrogen and ground to powder. Samples were resuspended in 1 ml of 50 mM sodium-phosphate buffer, extracted for 20 min on a lab shaker at 4 °C, and solid phase extraction was performed with 30 mg Oasis HLB columns equilibrated with 50 mM phosphate buffer, pH 3.0 as in ref. 52. Briefly, sample pH was adjusted to 3 with HCl, applied to the HLB column, washed twice with 2 ml 5% methanol, eluted with 2 ml 80% methanol and dried under nitrogen. Standards added were 25 ng 13C-IAA Cambridge Research Biochemicals) and indole-3-proprionic acid (Sigma). Calibration of indole-3-proprionic acid and 13C-IAA was calculated with 25 ng of oxindole-3-acetic acid (OlChemIm, Ltd., Olomouc, Czech Republic). Samples were resuspended in methanol and analysed by Liquid Chromatography-Multiple Reaction Monitoring-Mass Spectrometry (LC-MRM-MS) using an Agilent triple quad 6460. For each measurement, three samples were analysed. Results presented are means and s.d.

### Differential expression

A gene was considered to be expressed if its expression was >100, and differentially expressed if a *t*-test between two time points was significant at a *q* value of 0.05 after Benjamini–Hochberg false discovery rate correction^53^. For most analyses, we further restricted this list to include genes that were only twofold induced or repressed (>8,000 genes). More than 50% of probed genes (>12,000) were significantly expressed in the main LR time course. Further analyses were performed using Excel 2010 (Microsoft Corporation, Redmond, USA).

### Clustering

For each time course, we summarize the information contained in that profile by fitting a smoothing spline through the data. For details of smoothing splines, see ref. 54, p. 230. The basic idea is to fit a smooth curve through the data with a penalty on the roughness of the curve. The penalty is specified in the form of a multiplier of the integral of the squared second derivative of the curve in the objective function. We use the R function ‘smooth spline’ to fit the splines, and set the spar parameter to 0.4 (the multiplier described in the previous sentence is a monotone function of this parameter). We normalize each fitted spline to have mean 0 and variance 1 across the 18 time points of the experiment.

To cluster the normalized spline fits for differentially expressed genes in the LR time course data set, we use a method that combines hierarchical and *k*-means clustering. To cluster a set of several thousand genes, we proceed by breaking the data set-up into smaller blocks of 1,000 (plus 1 block containing <1,000 genes as the remainder). We cluster each block independently and then combine the centres of the different blocks to construct a set of centres on which we perform *k*-means clustering. The algorithm is as follows: calculate the spline fits for each gene and normalize to have mean 0 and variance 1; For a set of *N*=1,000 *B*+*R* genes, where *B* is the number of blocks of 1,000 and *R* is the number (<1,000) in the remainder, break the data set into *B* blocks of 1,000 genes and 1 block of R genes; For each of the (*B*+1) blocks, perform hierarchical clustering on the normalized splines using the Ward linkage and cutting the dendrogram at a height *h*_max_ to generate (*B*+1) sets of centres, where each set will in general contain a different number of centres; Combine the sets of centres and cluster using hierarchical clustering, again using the Ward linkage and cutting the dendrogram at a height *2* × *h*_max_ to generate an overall set of centres; Use *k*-means clustering on the data set using the overall set of centres from the previous step as the initial centres in the algorithm.

This algorithm is computationally very fast, even for several thousand genes, taking only a few minutes to run. Initially, we applied the above algorithm (with *h*_max_*=2)* to the differentially expressed transcription factors (TFs) (1,304 genes) and to the other differentially expressed genes (12,318 genes) independently. This yielded a clustering with 45 centres for the TFs, and 174 centres for the non-TFs. A total of 219 clusters seemed very large, and possibly unnecessarily complex. We therefore decided to compare each of the non-TF centres to each of the 45 TF centres. We used Euclidean distance between the vectors representing the centres as a measure of dissimilarity. We created a set of centres consisting of the 45 TF centres together with the non-TF centres that were at least a distance of 2 away from all of the 45 TF centres. We used the resulting set of 77 centres to perform *k*-means clustering of the entire set of differentially expressed genes. After obtaining the new centres following the *k*-means clustering, we summarized each cluster by taking the mean at each time point of each of its elements.

### Estimating the period for oscillatory gene clusters

After using the spline clustering method outlined in the previous section, we identified 17 of the 77 clusters whose mean time profiles looked oscillatory. To estimate the period of these clusters, we proceeded as follows: for each oscillatory cluster, fit a smoothing spline through the data points with a spar parameter of 0.1 (which corresponds to a low level of smoothing, since we have already done the smoothing to obtain the initial centres for the *k*-means clustering); Use the predict.smooth.spline function in R to estimate the first derivative of the fitted curve at a grid of 1,000 points over the range of the length of the experiment (54 h in our case); If the derivative is exactly 0 at any of the time points on the grid, then use those time points to index the peaks and troughs. Otherwise use the average of neighbouring time points between which the sign of the first derivative changes as the indices of the turning points; For each set of peaks and troughs (turning points), calculate the time differences between neighbouring turning points, average these differences and double to obtain an estimate of the period for a particular oscillatory cluster; This analysis gives periods ranging between 19.4 and 35.9 h, with six clusters showing periods within 1.5 h of 24 h (that is, between 22.5 and 25.5 h).

## Additional information

**How to cite this article:** Voß, U. *et al*. The circadian clock rephases during lateral root organ initiation in *Arabidopsis thaliana*. *Nat. Commun.* 6:7641 doi: 10.1038/ncomms8641 (2015).

## Supplementary Material

Supplementary Figures, Tables, Methods and References Supplementary Figures 1-5, Supplementary Tables 1-2

Supplementary Data 1 Cluster to gene mappings

Supplementary Data 2 Circadian analysis of lateral root transcriptome

Supplementary Data 3 Circadian analysis of auxin related genes
