# Supplementary material for: The circadian clock rephases during lateral root organ initiation in Arabidopsis thaliana
Source: Nat Commun. 2015 Jul 6;6:7641. doi: 10.1038/ncomms8641 (PMC4506504; doi:10.1038/ncomms8641)
Supplement: Supplementary Figures, Tables, Methods and References — Supplementary Figures 1-5, Supplementary Tables 1-2 [file ncomms8641-s1.pdf]

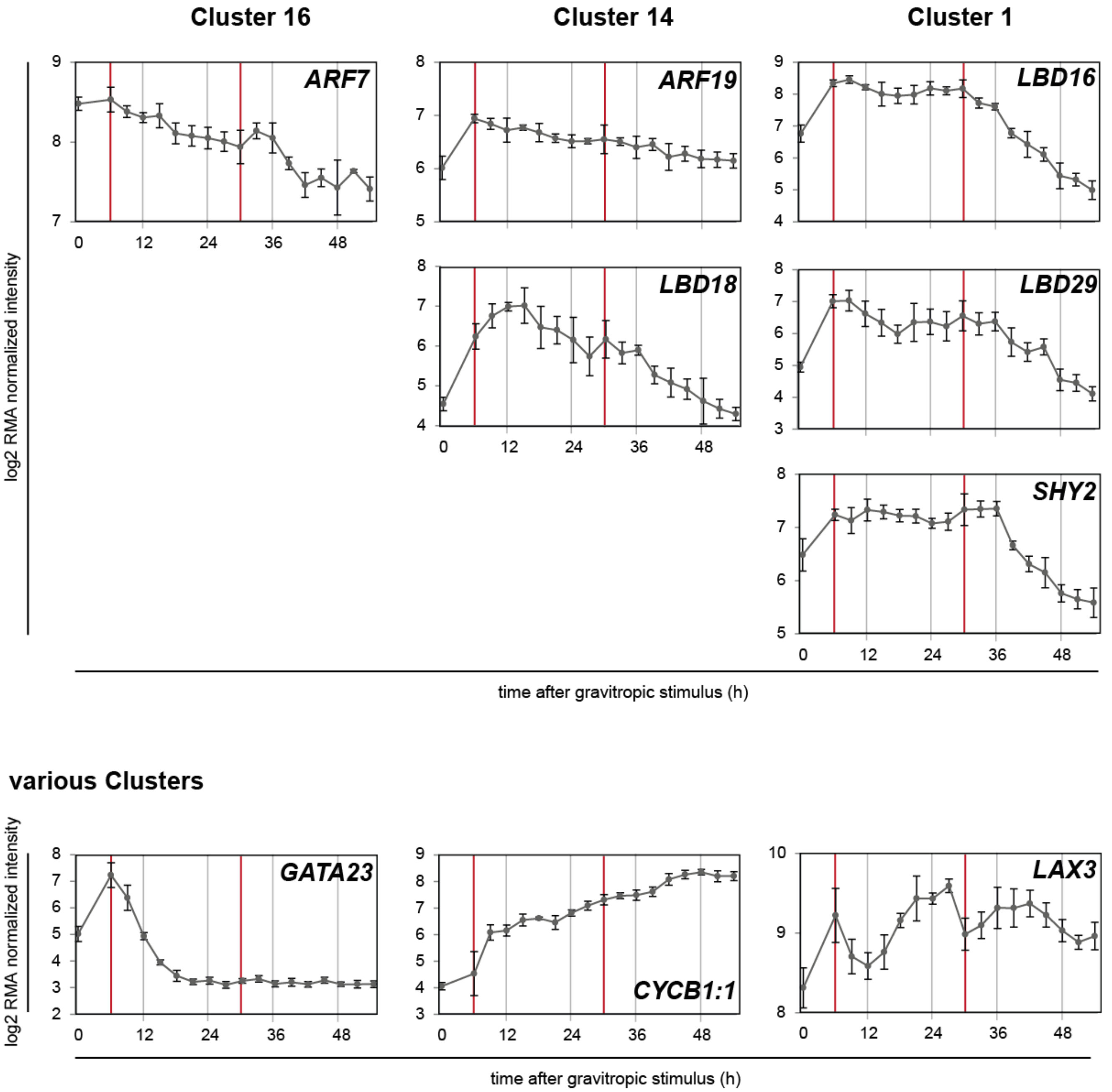

Supplementary Figure 1. Expression profiles of lateral root regulatory genes. mRNA expression profiles of the lateral root regulator genes *ARF7*, *ARF19, LBD18*, *LBD16*, *LBD29*, *SHY2*, *GATA23*, *CYCB1;1* and *LAX3* in the LR transcriptomic timecourse dataset. Expression intensities from the LR microarray data are on a $\log_2$ scale. Red vertical bars times of peak *TOC1* expression. All seedlings were grown in constant light conditions, without previous entrainment. Error bars are the mean of all 4 replicates plus or minus two standard errors. Cluster numbers are indicated above expression profiles.

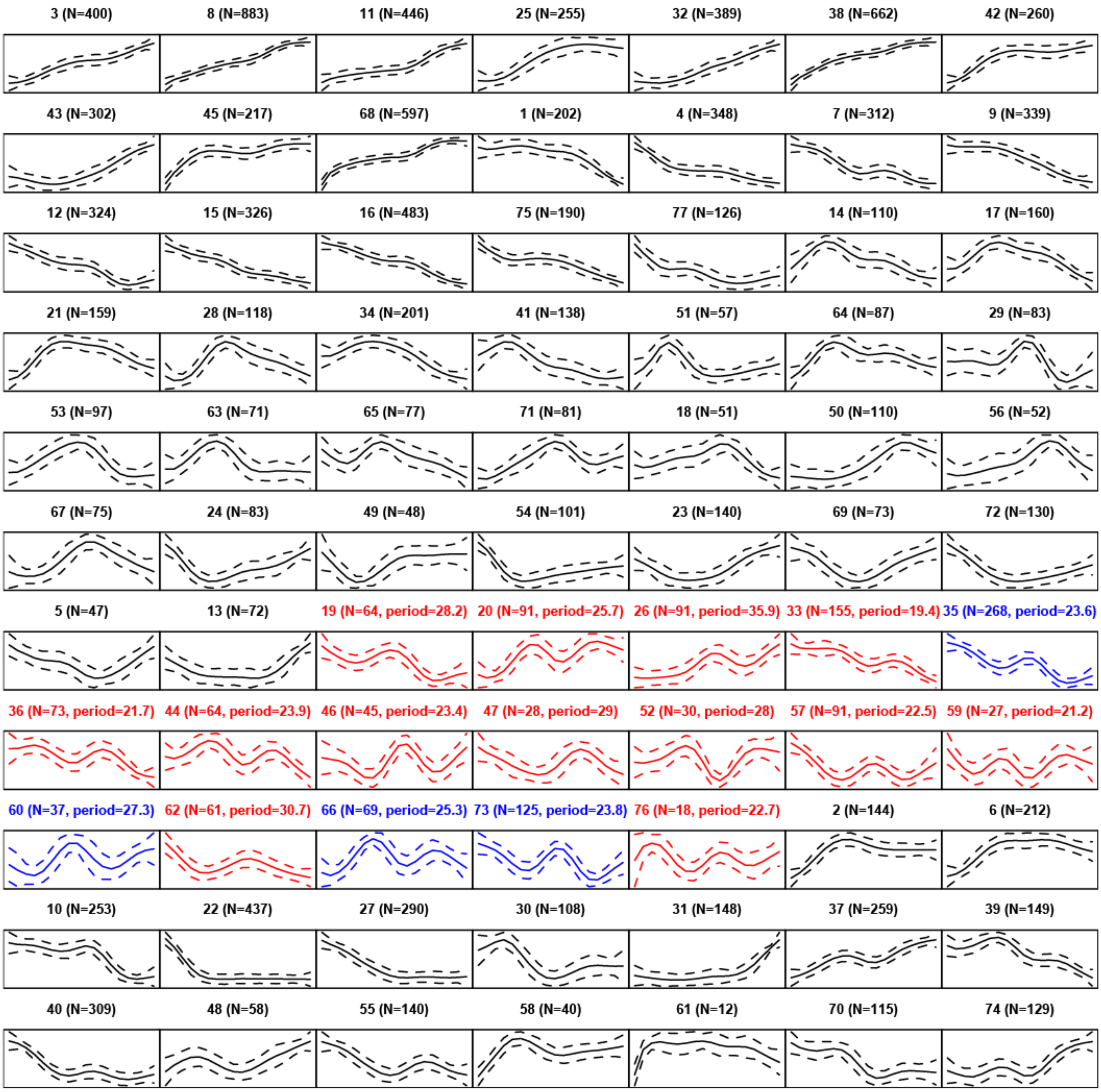

Supplementary Figure 2. Expression-based clustering of the lateral root transcriptomic dataset. A combination of hierarchical and *k*-means clustering was used to cluster the expression profiles of the differentially expressed genes into 77 clusters. The oscillatory clusters are coloured, as in Figure 1E. The cluster mean profiles give an overview of the shapes of expression patterns observed across the whole data set. Expression intensities from the LR microarray data are on a $\log_2$ scale. All seedlings were grown in constant light

conditions, without previous entrainment. The mean of the gene expression is given by the solid line and the dotted lines show the mean plus or minus two standard errors. Cluster numbers and size (N = gene number) are indicated above expression profiles.

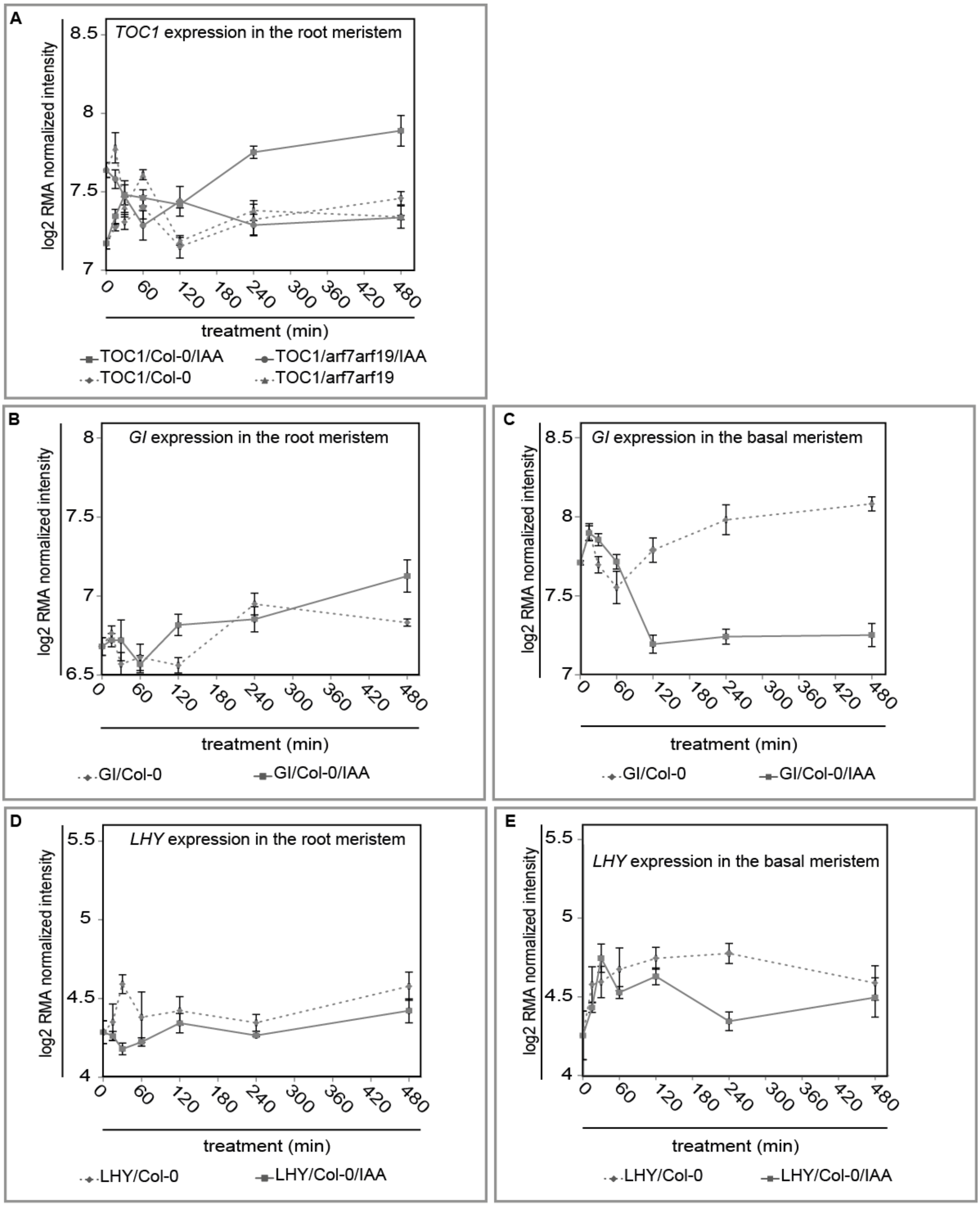

Supplementary Figure 3. Circadian clock gene expression in response to auxin. *TOC1, GI* and *LHY* mRNA pattern changes in the root tissue in response to auxin treatment (1µM IAA) in Col-0 as well as *arf7arf19* mutant plants. *TOC1* **(A),** *GI* **(B)** transcript abundance in

the root meristem **(B)** and in the basal meristem **(C);** *LHY* **(D)** transcript abundance in the root meristem **(B)** and in the basal meristem **(C)** in response to auxin treatment in *Col-0* seedlings.**(A-C)** Expression intensities are on a $\log_2$ scale. Error bars are the mean of all three replicates plus or minus two standard errors.

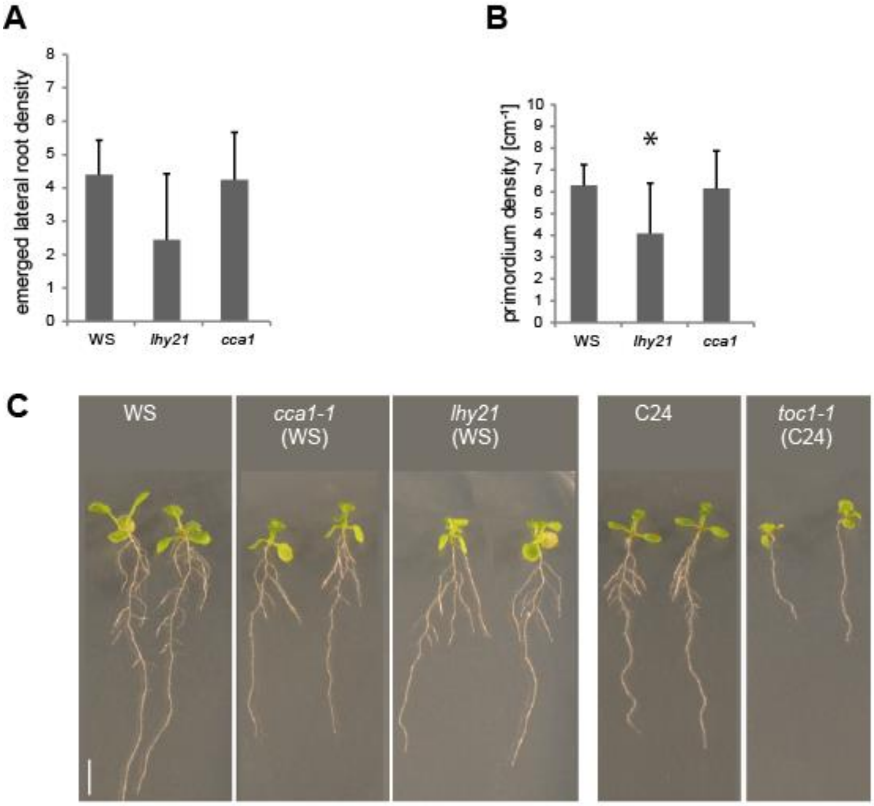

Supplementary Figure 4. Lateral root phenotypes of circadian clock mutants. All seedlings were grown in constant light conditions, without previous entrainment. N=20 for all experiments. Error bars indicate standard deviation; asterisks indicate a significant difference with T-Test ($p \leq 0.05$) from wildtype. **(A-B)** Emerged lateral root **(A)** and primordium **(B)** density of *cca1* and *lhy1* seedlings and their corresponding wildtype C24 in cm^{-1}. **(C)** Representative images of circadian clock mutant seedlings and their corresponding wildtypes (*WS* for *cca1* and *lhy1* and C24 for *toc1-1*). Scale bar = 0.5cm.

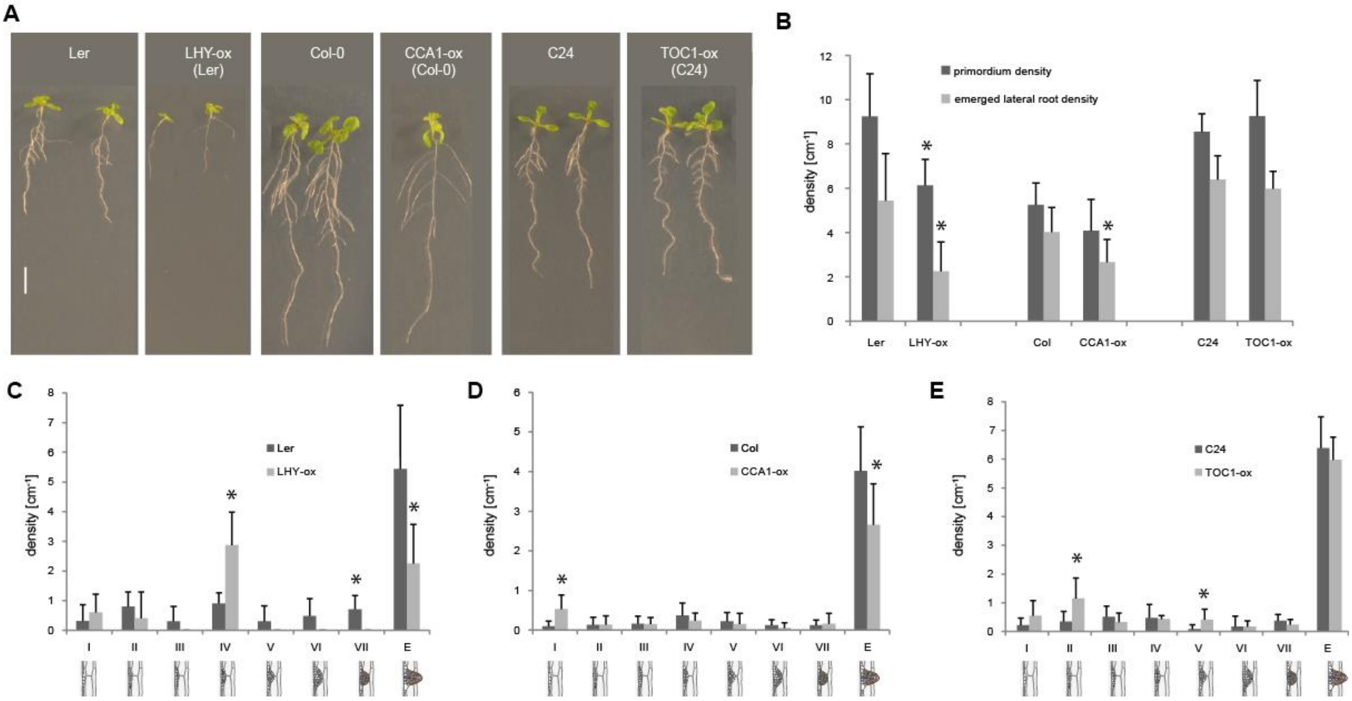

Supplementary Figure 5. Lateral root phenotypes of circadian clock mutants. All seedlings were grown in constant light conditions, without previous entrainment. N=20 for all experiments. Error bars indicate standard deviation; asterisks indicate a significant difference with T-Test ($p \leq 0.05$) from wildtype. **(A)** Representative images of circadian clock gene overexpressing lines and their corresponding wildtypes (*Ler* for *LHY-OX, Col0* for *CCA1-OX* and C24 for *TOC1-OX*). Scale bar = 0.5cm. **(B)** Emerged lateral root and primordium density of circadian clock gene overexpressing lines and their corresponding wildtypes in cm^{-1}. **(C-E)** 10 day old seedlings were fixed and the primordium density of different developmental stages (stage I to emerged (E)) of wildtype and circadian clock gene overexpressing lines in cm^{-1} was determined.

timepoint (after gravistimulus)	plates put in growthchamber (LL)		gravitropic stimulus		sampling of root bends	
	day	time	day	time	day	time
0h	1	7	-	-	4	16
6h	1	7	4	7	4	13
9h	1	7	4	7	4	16
12h	1	7	4	7	4	19
15h	1	16	4	16	5	7
18h	1	16	4	16	5	10
21h	1	16	4	16	5	13
24h	1	16	4	16	5	16
27h	1	16	4	16	5	19
30h	2	1	5	1	6	7
33h	2	1	5	1	6	10
36h	2	1	5	1	6	13
39h	2	1	5	1	6	16
42h	2	1	5	1	6	19
45h	2	10	5	10	7	7
48h	2	10	5	10	7	10
51h	2	10	5	10	7	13
54h	2	10	5	10	7	16

Supplementary Table 1. Timings of Sampling. The sampling timetable for the LR transcriptomic dataset gives details of seedling growth, gravitropic induction and sampling. Day indicates consecutive days of experiment, starting with day 1 (where first set of seedlings is transferred to growth chamber). Time: time of day at which experiment is performed. This was applied to all four replicates.

Primer name	sequence
qACT2F	ccgctctttctttccaagc
qACT2R	ccggtaccattgtcacacac
Q-CCA1-s	aaaaagtgtcgcatcctgaga
Q-CCA1-as	gaacagttgtcttcctgcagagt

Supplementary Table 2. Primer sequences. The Table gives the sequences of the primers used for RTq-PCRs shown in Figure 3.
